# 312. Impact of Patient Subgroups on the Efficacy of Ceftazidime-Avibactam (CZA) versus Ceftolozane-Tazobactam (C/T) for Multidrug-resistant (MDR) *Pseudomonas aeruginosa* infections in the United States (CACTUS)

**DOI:** 10.1093/ofid/ofae631.102

**Published:** 2025-01-29

**Authors:** Michael P Veve, jason M Pogue, Lilian M Abbo, Renee Ackley, Samuel L Aitken, Benjamin Albrecht, Ahmed Babiker, Rachel Burgoon, Kimberly C Claeys, Brooke Curry, Kate DeSear, Jason C Gallagher, Esther Golnabi, Sarah B Green, Alan E Gross, Emily L Heil, Krutika M Hornback, Keith S Kaye, Trieu-Vi Khuu, Megan Klatt, Ellen G Kline, Ryan C Kubat, Wesley D Kufel, Jae Hyoung Lee, Alexander Lepak, Conan MacDougall, Anjali Majumdar, Amy Mathers, Erin K McCreary, William R Miller, Marguerite Monogue, W Justin Moore, Shannon Olson, Jessica Oxer, Jeffrey C Pearson, Christine Pham, Paulette Pinargote, Christopher Polk, Michael J Satlin, Sarah W Satola, Sunish Shah, Pranita Tamma, Truc Cecilia Tran, David van Duin, Mollie VanNatta, Ana Vega, Venugopalan Veena, Walaiporn Wangchinda, Lucy S Witt, Ryan K Shields

**Affiliations:** Eugene Applebaum College of Pharmacy and Health Sciences, Detroit, Michigan; University of Michigan, College of Pharmacy, Ann Arbor, MI; University of Miami Miller School of Medicine, Jackson Health System, Aventura, FL; Atrium Health, Charlotte, North Carolina; Michigan Medicine, Ann Arbor, MI; Emory University Hospital, Atlanta, Georgia; Emory University, Atlanta, GA; Medical University of South Carolina, Charleston, South Carolina; University of Maryland Baltimore, Baltimore, Maryland; University of Illinois at Chicago, Chicago, Illinois; UF Health Shands Hospital, Gainesville, FL; Temple University, Philadelphia, PA; UT Southwestern, Dallas, Texas; Emory University Hospital, Atlanta, Georgia; University of Illinois, Chicago, IL; University of Maryland School of Pharmacy, Baltimore, MD; MUSC Health, Mt Pleasant, SC; Rutgers Robert Wood Johnson Medical School, New Brunswick, NJ; University of North Carolina, Chapel Hill, North Carolina; The University of Kansas Health System, KS; University of Pittsburgh, Pittsburgh, Pennsylvania; University of Kansas, Kansas City, Kansas; Binghamton University School of Pharmacy Sciences, Binghamton, NY; Johns Hopkins, Baltimore, Maryland; University of Wisconsin School of Medicine and Public Health, Madison, Wisconsin; University of California San Francisco, San Francisco, CA; Rutgers-Robert Wood Johnson Medical School, New Brunswick, New Jersey; University of Virginia, Charlottesville, VA; University of Pittsburgh Medical Center, Pittsburgh, PA; Houston Methodist Research Institute, Houston, TX; University of Texas Southwestern Medical Center, Dallas, TX; Northwestern Medicine, Chicago, IL; Sinai-Grace Hospital Detroit Medical Center, Detroit, Michigan; Weill Cornell Medicine, New York, New York; Brigham and Women's Hospital, Boston, MA; University of California, Los Angeles; David School of Medicine/University of California, Los Angeles, Los Angeles, California; Lousiana State University Health Shreveport, Bossier City, Louisiana; Atrium Health, Charlotte, North Carolina; Weill Cornell Medicine, New York, New York; Emory University School of Medicine, Division of Infectious Diseases, Atlanta, Georgia; Antibiotic Management Program, UPMC Presbyterian Hospital, Pittsburgh, PA, Pittsburgh, Pennsylvania; Johns Hopkins School of Medicine, Baltimore, MD; Houston Methodist Research Institute, Houston, TX; University of North Carolina at Chapel Hill, Chapel Hill, NC; Ochsner LSU Health Shreveport, Shreveport, Louisiana; Jackson Memorial Hospital, Miami, Florida; University of Florida, College of Pharmacy, Gainesville, Florida; University of Michigan College of Pharmacy, Ann Arbor, Michigan; Emory University, Atlanta, GA; University of Pittsburgh, Pittsburgh, Pennsylvania

## Abstract

**Background:**

CACTUS is a retrospective, matched, multicenter study comparing the efficacy of C/T and CZA for treatment of bacteremia or pneumonia due to MDR *P. aeruginosa*. We found that treatment with C/T resulted in higher rates of clinical success compared to CZA after controlling for baseline differences between cohorts. The objective of this study is to determine the impact of patient subgroups on the overall findings.

**Figure:**
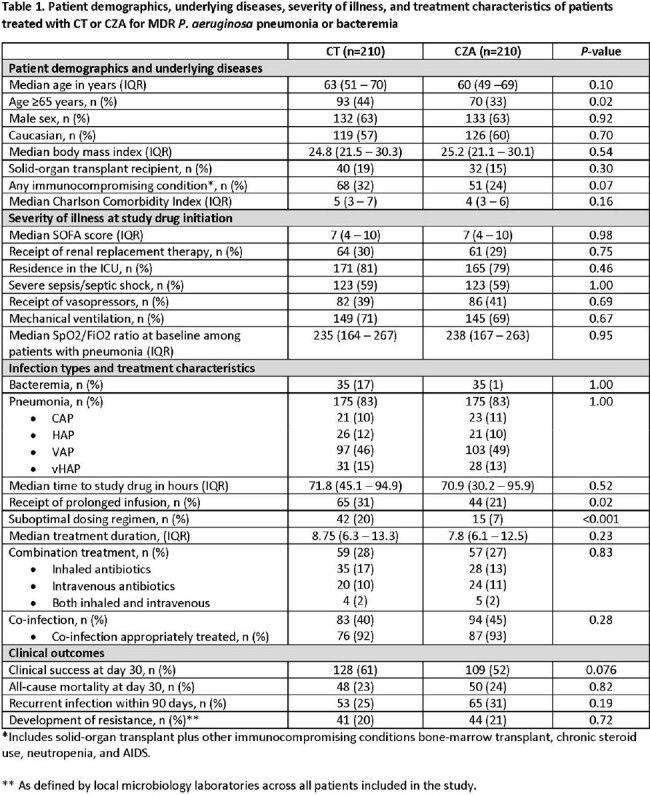

**Methods:**

C/T and CZA patients were matched 1:1 within each study site based on severity of illness, infection type, and time to treatment initiation. The primary outcome was clinical success at day 30, defined as survival, resolution of signs/symptoms of infection with the intended treatment course, and absence of recurrent infection. For subgroups of interest, the proportion with clinical success in each group was compared using an unadjusted odds ratio (OR) and 95% confidence interval.

**Figure:**
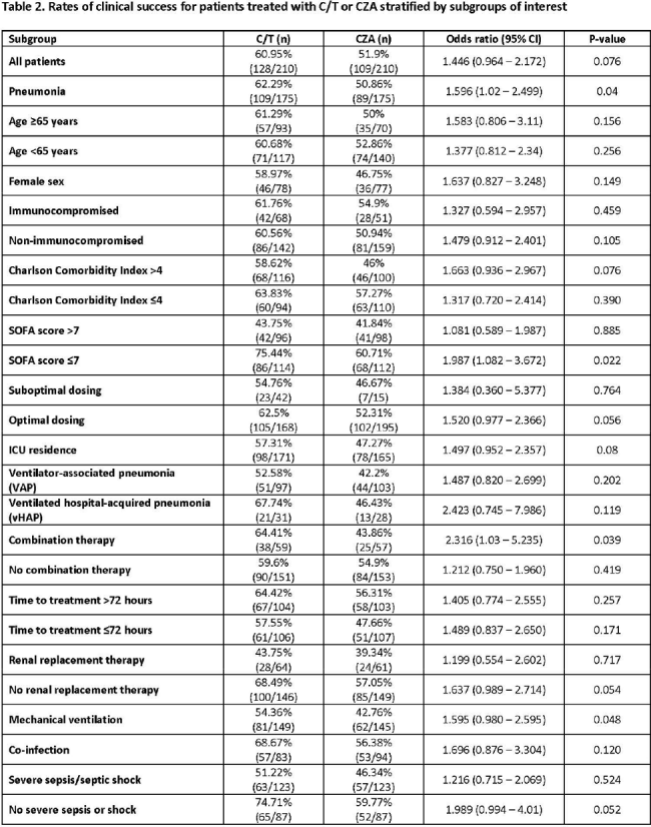

**Results:**

Among 420 cases from 28 sites, 60.95% (128/210) of C/T- and 51.9% (109/210) of CZA-treated patients experienced clinical success. Groups were overall well-balanced by matching; however, CT-treated patients were older, more likely to have immunocompromising conditions, and to receive suboptimal dosing (**Table 1**). After conditional logistic regression, the adjusted OR for clinical success with C/T treatment was 1.97 (95% CI: 1.11 – 3.49; *P*=0.02).

Unadjusted OR for patient subgroups are listed in **Table 2.** Overall, the effect size was consistent across all subgroups. Key factors are highlighted in **Figure 1.** Significantly higher rates of clinical success were found for treatment with C/T compared to CZA for patients with pneumonia, SOFA ≤7, mechanical ventilation, and optimal dosing**. Table 3** displays comparative 30-day mortality rates. Higher mortality rates were identified in both C/T- and CZA-treated patients who were critically-ill, received suboptimal dosing, and were treated within 72 hours of index culture (**Table 3**); rates did not vary by treatment.

**Figure:**
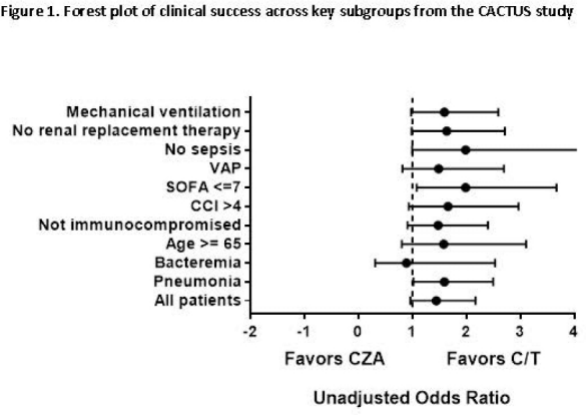

**Conclusion:**

The consistency of the results in this subgroup analysis of CACTUS reinforces the primary finding of higher rates of clinical success for patients with MDR *P. aeruginosa* pneumonia or bacteremia treated with C/T compared to CZA.

**Figure:**
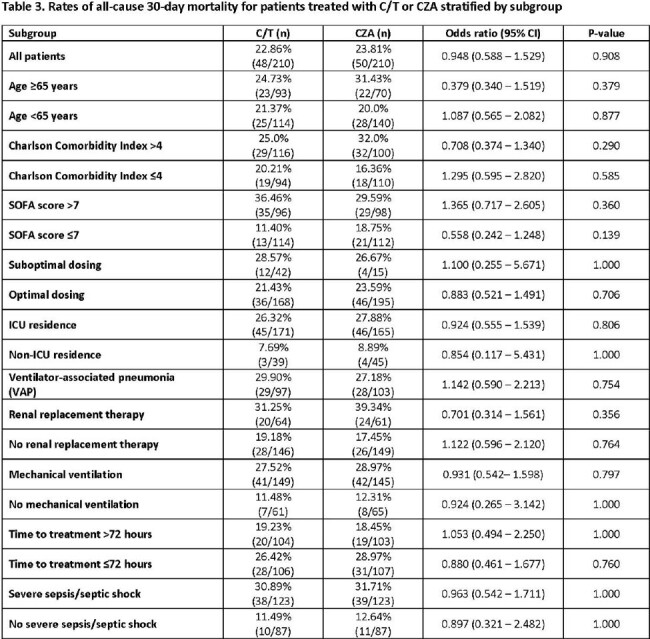

**Disclosures:**

**jason M. Pogue, PharmD**, Entasis: Advisor/Consultant|GSK: Advisor/Consultant|Melinta: Advisor/Consultant|Melinta: Grant/Research Support|Merck: Advisor/Consultant|Merck: Grant/Research Support|Shionogi: Advisor/Consultant|Shionogi: Grant/Research Support|Venatorx: Advisor/Consultant **Samuel L. Aitken, PharmD, MPH**, Basilea: Advisor/Consultant|bioMerieux: Advisor/Consultant|Melinta: Advisor/Consultant|Shionogi: Advisor/Consultant **Ahmed Babiker, MBBS**, Beckman Coulter Inc.: Advisor/Consultant **Kimberly C. Claeys, PharmD, PhD**, bioMérieux: Advisor/Consultant|bioMérieux: Honoraria **Kate DeSear, PharmD, BCIDP, AAHIVP, FIDSA**, AbbVie Inc: Advisor/Consultant|Basilea Pharmaceutica: Advisor/Consultant|GSK: Advisor/Consultant|La Jolla (Entasis): Advisor/Consultant|Melinta Therapuetics: Advisor/Consultant **Alan E. Gross, PharmD**, Becton Dickinson Co: Advisor/Consultant **Keith S. Kaye, MD, MPH**, Allecra: Advisor/Consultant|CARB-X: Advisor/Consultant|GSK: Advisor/Consultant|Merck: Advisor/Consultant|Shionogi: Advisor/Consultant|Spero: Advisor/Consultant **Wesley D. Kufel, Pharm.D., BCPS, BCIDP**, Merck & Co.: Grant/Research Support|Shionogi, Inc: Grant/Research Support **Conan MacDougall, PharmD, MAS**, Merck: Grant/Research Support **Erin K. McCreary, PharmD**, Abbvie: Advisor/Consultant|Basilea: Advisor/Consultant|Ciadara: Advisor/Consultant|Entasis: Advisor/Consultant|Ferring: Advisor/Consultant|GSK: Advisor/Consultant|GSK: Honoraria|Melinta: Advisor/Consultant|Merck: Advisor/Consultant|Pfizer: Honoraria|Shionogi: Advisor/Consultant|Shionogi: Honoraria **William R. Miller, M.D.**, Merck: Grant/Research Support|UptoDate: Royalties **Jeffrey C. Pearson, PharmD**, inflarx: Advisor/Consultant **Michael J. Satlin, MD**, AbbVie: DSMB participant|bioMerieux: Grant/Research Support|Merck: Grant/Research Support|Selux Diagnostics: Grant/Research Support|SNIPRBiome: Grant/Research Support **David van Duin, MD, PhD**, Merck: Advisor/Consultant|Merck: Grant/Research Support|Pfizer: Advisor/Consultant|Qpex: Advisor/Consultant|Roche: Advisor/Consultant|Shionogi: Advisor/Consultant|Shionogi: Grant/Research Support **Ryan K. Shields, PharmD, MS**, Allergan: Advisor/Consultant|Cidara: Advisor/Consultant|Entasis: Advisor/Consultant|GSK: Advisor/Consultant|Melinta: Advisor/Consultant|Melinta: Grant/Research Support|Menarini: Advisor/Consultant|Merck: Advisor/Consultant|Merck: Grant/Research Support|Pfizer: Advisor/Consultant|Roche: Grant/Research Support|Shionogi: Advisor/Consultant|Shionogi: Grant/Research Support|Utility: Advisor/Consultant|Venatorx: Advisor/Consultant|Venatorx: Grant/Research Support

